# Alveolar epithelial-to-mesenchymal transition in scleroderma interstitial lung disease: Technical challenges, available evidence and therapeutic perspectives

**DOI:** 10.1177/23971983231181727

**Published:** 2023-06-20

**Authors:** Enrico De Lorenzis, Christopher William Wasson, Francesco Del Galdo

**Affiliations:** 1 Leeds Institute of Rheumatic and Musculoskeletal Medicine, University of Leeds, Leeds, UK; 2 Division of Rheumatology, Catholic University of the Sacred Heart, Fondazione Policlinico Universitario A. Gemelli IRCCS, Rome, Italy; 3 NIHR Leeds Biomedical Research Centre, Leeds Teaching Hospitals NHS Trust, Leeds, UK

**Keywords:** Scleroderma-related interstitial lung disease, epithelial-to-mesenchymal transition, systemic sclerosis, transitional research

## Abstract

The alveolar epithelial-to-mesenchymal transition is the process of transformation of differentiated epithelial cells into mesenchymal-like cells through functional and morphological changes. A partial epithelial-to-mesenchymal transition process can indirectly contribute to lung fibrosis through a paracrine stimulation of the surrounding cells, while a finalized process could also directly enhance the pool of pulmonary fibroblasts and the extracellular matrix deposition. The direct demonstration of alveolar epithelial-to-mesenchymal transition in scleroderma-related interstitial lung disease is challenging due to technical pitfalls and the limited availability of lung tissue samples. Similarly, any inference on epithelial-to-mesenchymal transition occurrence driven from preclinical models should consider the limitations of cell cultures and animal models. Notwithstanding, while the occurrence or the relevance of this phenomenon in scleroderma-related interstitial lung disease have not been directly and conclusively demonstrated until now, pre-clinical and clinical evidence supports the potential role of epithelial-to-mesenchymal transition in the development and progression of lung fibrosis. Evidence consolidation on scleroderma-related interstitial lung disease epithelial-to-mesenchymal transition would pave the way for new therapeutic opportunities to prevent, slow or even reverse lung fibrosis, drawing lessons from current research lines in neoplastic epithelial-to-mesenchymal transition.

## Introduction

Systemic sclerosis (SSc) is the condition associated with higher mortality among rheumatic diseases, mainly as a direct or indirect consequence of lung involvement.^1^ Scleroderma-related interstitial lung disease (SSc-ILD) is a severe fibrotic and vascular complication characterized by an excessive deposition of extracellular matrix (ECM) that leads to impaired gas exchanges and increased vascular resistance of the pulmonary circulation.

Two main distinct histologic patterns have been recognized in SSc-ILD, namely nonspecific interstitial pneumonia (NSIP) and usual interstitial pneumonia (UIP). The NSIP pattern is associated with more than two-thirds of SSc-ILD patients and is characterized by a variable extent of alveolar septa thickening, lymphocytic interstitial infiltrate, fibrosis and neo-vascularization.^2^ The UIP pattern is conversely observed in a smaller proportion of patients and replicates the abnormalities of idiopathic pulmonary fibrosis (IPF)^3^ with fibrotic areas mainly organized into foci of fibroblasts, myofibroblasts and a few inflammatory cells adjacent to normal parenchyma.^3^

Despite these substantial histologic dissimilarities, it is still substantially unknown whether different triggers or biological pathways could lead to distinct parenchymal structural changes. Nevertheless, crucial differences between NSIP and UIP in SSc have not been conclusively recognised in terms of epidemiology, progression rate, treatment response or related mortality.^4^

Multiple sources have been demonstrated or postulated for the hyperactive fibroblast pool involved in SSc-ILD ECM deposition, including activation of quiescent resident tissue fibroblasts, recruitment of circulating fibrocytes or transformation of pericytes, endothelial cells and alveolar epithelial cells (AECs).^5^

The epithelial-to-mesenchymal transition (EMT) is a process of gradual transformation of fully differentiated epithelial cells towards mesenchymal-like cells through functional and morphological changes.^6^ While physiological EMT events take place during organogenesis (type 1), pathological EMT has been linked to abnormal healing processes in fibrosis (type 2) and carcinoma metastatic invasion (type 3).^7^ Of note, the EMT is a gradual, reversible and sometime incomplete process, so a full transformation of epithelial cells into fibroblast or myofibroblast could be often non-finalized. It is crucial to stress the concept of partial activation of an EMT programme because it could explain some conflictual experimental data, without disproving a significant contribution to fibrogenesis and inflammation through the paracrine stimulation of the surrounding cells.^8^

## EMT within alveolar epithelium

### Distal airways as a predisposing context for EMT

The alveolar microenvironment plays an important role in the induction of EMT. AECs are considered dynamic cytotypes in a strategic interface location that can adapt to physiological and pathological stimuli and reprogram themselves through a set of transcription factors and microRNAs. The functional unit of the lung – the alveolus – is composed by the alveolar epithelium and the alveolar septa, which contain the pulmonary capillaries participating in gas exchange and some connective tissue. The alveolar epithelium layer is made up of two types of AECs. Type 1 AECs are flat cells that regulate gas transport, fluid balance among the alveoli and surfactant production by type 2 AECs through mechanical stimulation. Conversely, type 2 AECs are progenitor cells that differentiate into type 1 AECs, produce surfactant and can also present antigens to immune cells.^9^

**Figure 1.**
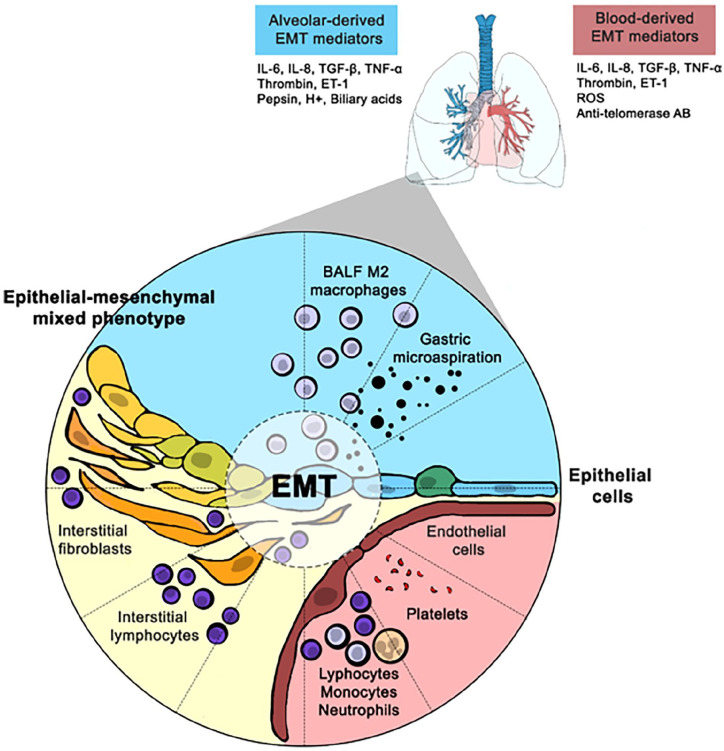
Putative mechanisms of EMT alveolar induction in SSc-ILD patients. AB: antibody; BALF: bronchoalveolar lavage fluid; EMT: epithelial-to-mesenchymal transition; ET: endothelin; IL: interleukin; ILD: interstitial lung disease; ROS: reactive oxygen species; SSc: systemic sclerosis; TGF- β: transforming growth factor β; TNF-α: tumour necrosis factor α.

This peculiar anatomical organization is functional to gas exchanges and directly exposes AECs to alveolar, interstitial and intravascular environments. These cells are consequently in a close relationship with mesenchymal interstitial cells, endothelial cells and immune cells dwelling in the alveolar and interstitial spaces, most of which are macrophages in both physiological and pathological conditions^10^ (Figure 1).

Although type 2 AECs are postulated to be the main responsible for both EMT processes and type AEC differentiation, it is also known that type 1 can differentiate back into type 2 cells suggesting the participation of all epithelial cells in the mesenchymal transition process.^11^

### Methods and caveats of direct alveolar EMT assessment strategies

The easiest and most used method to demonstrate the occurrence of EMT is to report the cellular co-expression of mesenchymal and epithelial markers through western blotting, immunohistochemistry or flow cytometry. Mesenchymal markers include surface molecules such as N-Cadherin, cytoskeletal components such as vimentin, alpha-Smooth Muscle Actin and fibroblast-specific protein 1 and ECM components such as fibronectin or type 1 collagen. Nevertheless, transcription factors such as Snail, Slug, ZEB-1, ZEB-2, Twist and β-catenin could also be tested as markers reflecting the activation of four main interconnected pathways involved in the EMT process, namely canonical and non-canonical transforming growth factor β1 (TGF-β1), Wnt-Catenin and Notch signalling.^12^

This approach based on marker co-expression has shown some limitations the first of which is the intention to record a dynamic EMT process through a static biological picture. Furthermore, all these markers are liable to a variable degree of specificity in different types of cells and a simultaneous evaluation of a group of them is, therefore, necessary.^13^ Finally, in the case of a complete EMT, epithelial markers are not expressed anymore, and the epithelial origin of a differentiated fibroblast must be unequivocal also in the more complex in vivo systems.

Single cell RNA sequencing, genetic fate tracking and intravital microscopy are new techniques of real-time EMT assessment that aim to overcome the limitations described above. Single cell RNA sequencing is a genomic approach for the detection and quantitative analysis of messenger RNA molecules in specific populations of cells, including AECs. Single cell RNA sequencing could evaluate the transcriptome as a proxy of the proteome to explore the co-expression of mesenchymal and epithelial markers but still presents some standardization and interpretation challenges^14^ and still provides a static description of the cellular processes. Lineage tracing relies on site-specific recombinase-mediated DNA excision to switch the expression of some reporter molecules and allow therefore the identification of the cellular progeny arising from an individual cell.^15^ It remains technically challenging and is prone to criticism about choices, so it sometimes provided conflicting evidence.^16–18^ Intravital microscopy – that is, microscope imaging of live animals – is an increasingly adopted technique that has been mainly applied to EMT evaluation in neoplastic lesions. Of note, this kind of real-time evaluation is thought to be applied to the lungs because of their enclosed position within the body, respiratory movements and physical impact of the heartbeat.^19^

## Alveolar EMT in SSC-ILD patients and preclinical models

The stronger evidence of EMT occurrence is through the direct analysis of human biopsy lung samples but indirect evidence is commonly derived from cell cultures and animal models of SSc-ILD. Of note, also the bronchoalveolar lavage fluid (BALF) analysis is currently available as a less-invasive tool to directly assess the alveolar environment as a surrogate of lung biopsies.^20^ Although the BALF analysis is not able to directly characterize the lung parenchyma, it indeed explores in terms of cellular and acellular components the biological compartment that is in closer contact with AECs.

By the time of this review, EMT has never been assessed though epithelial and mesenchymal marker co-expression in lung samples of patients with SSc as it was previously done in IPF^16,21^ or lung-transplanted patients.^22^ One of the reasons is that surgical lung biopsy is seldom performed in SSc compared to IPF or transplanted patients since current evidence did not support its routinary diagnostic or prognostic use in SSc-ILD. A single RNA-assessment study, including the sequencing of AEC transcriptome, have indicated the simultaneous transcription of mesenchymal and epithelial markers in a specific subpopulation of aberrant basaloid cells in lung biopsies. Although this observation clearly supports an EMT occurrence, it was derived from a small population of patients with UIP pattern and long-stage disease and therefore preclude wider inferences on SSc-ILD pathophysiology.^23^

An in vivo active pathophysiological contribution of AECs to SSc-ILD is anyway clearly indicated by the increased serum level of AEC-secreted glycoproteins, such as surfactant protein D (SP-D) and Krebs von den Lungen 6 (KL-6),^24^ and by functional nuclear medicine studies showing that alveolar epithelium permeability is increased in SSc-ILD and predicts a worse prognosis.^25,26^

Given the mentioned limitations, lung epithelium cell cultures are by far the most diffuse source of biological information on EMT in SSC-ILD.^27^ As detailed below, inferences on SSc-ILD pathophysiology were driven from experimental conditions that reproduced biological or chemical stimuli that are over-represented in SSc lung^28^ since direct exposure to patient serum or BALF was never tested until now, and AEC lines are hard to develop from lung biopsies of SSc patients. Of note, many inferences have been driven from immortalized or tumour-derived cell lines because of technical advantages and relatively low costs, although the related results should be cautiously interpreted since EMT is known to be fostered by immortalization and cancer genesis.^29,30^

In addition to the mentioned in vitro studies, bleomycin-induced lung fibrosis is an extensively used and informative animal model of lung fibrosis in vivo in which EMT has been proven to occur also using lineage tracking methods.^17,31,32^ Although this model has been applied to both IPF and SSc-ILD evaluation, it is by far closer to the latter. The proximity of bleomycin-induced lung fibrosis to SSc-ILD is related to different reasons. First, the histology pattern shows pulmonary vascular endothelium damage at early stages (in case of intravascular, intraperitoneal or subcutaneous administration), followed by inflammation infiltration and homogeneous secondary fibrotic changes.^33^ Second, bleomycin-exposed animals often develop seropositivity to antinuclear antibodies (ANAs), anti-topoisomerase-I, anti-U1 RNP and anti-histone antibodies.^34^ Finally, the role of immune response is so striking that bleomycin-induced lung damage can improve after corticosteroid and mycophenolate mofetil administration.^35,36^

### Microvascular dysfunction as EMT-inducer in SSc-ILD

Structural and functional affection of microcirculation are milestones in the pathogenesis of SSc that are known to occur early in the natural history of the disease. These abnormalities are the presumptive consequence of multiple processes such as infections, immune-mediated cytotoxicity, anti-endothelial antibodies,^37^ or ischaemia-reperfusion^38^ and are manifested as aberrant vasomotor tone and Nitric Oxide metabolism, intravascular coagulation, leucocyte diapedesis, barrier permeability and possibly a direct contribution to fibrosis via endothelial-to-mesenchymal transition.^39,40^

A causal relationship between microcirculation abnormalities and pulmonary fibrosis could be driven by the clinical association of SSc-ILD with digital ulcers,^41^ capillaroscopy structural changes,^42^ anti-endothelium antibody positivity^43^ and increased serum markers of endothelial dysfunction such as E-selectin, P-selectin, von Willebrand Factor Antigen and ICAM1.^44,45^ Furthermore, the available BALF characterization data consistently show increased levels of endothelin-1^46^ and thrombin^47^ in SSc-ILD patients. Of note, even if endothelial cells are known to be major players in the synthesis or activation of the above paracrine mediators, these can also derive from epithelial cells, macrophages or mesenchymal cells as a part of a complex cellular crosstalk.^48,49^

The theory of a mechanistic relationship between microvascular disease and EMT is also supported by the in vitro effect of the main mediators of endothelial dysfunction on AECs. Oxidative stress,^50–52^ NO deficiency,^53^ endothelin-1^54^ and thrombin^55^ are all demonstrated to be EMT inducers in cellular or animal models of ILD.

### Inflammation as EMT-inducer in SSc-ILD

In SSc-ILD, immune activation is a main driver of fibrosis compared to the idiopathic forms as suggested by auto-antibody negativity and the relatively reduced presence of immune cells in IPF lung biopsies.^56^ The role of immune cells is highlighted by the numerous correlations of BALF cellularity with respiratory symptoms, lung imaging, pulmonary function and even mortality in SSc-ILD patients.^57^ The macrophages are the most represented immune cell type in the alveolar space and interstitium, and their circulating precursors are prone to develop an alternative M2 activation profile with pro-fibrotic properties^58,59^ and an expected promotion of EMT.^60^ The specific role of lymphocytes in the EMT process has been poorly defined despite the known efficacy of mycophenolate mofetil and rituximab in the treatment of this lung complication.^61^

In addition to BALF cells, some alveolar cytokines have been reported to be hyper-represented in BALF of SSc-ILD patients and show a correlation of their level with the severity of lung impairment. Of note, alveolar immune cells must be considered only one of the stakeholders involved in cytokine production in response to a plethora of biological, chemical or even mechanical stimuli. Pulmonary mesenchymal, endothelial and epithelial cells are indeed proven to contribute to the definition of the alveolar immune microenvironment in a series of self-sustained paracrine circles.

TGF-β, interleukin (IL)-6, tumour necrosis factor α (TNF-α) and IL-8 are the cytokines more consistently reported to be abnormal and associated with alveolitis in SSc-ILD. TGF-β1 has been implicated as a pivotal inducer of lung fibrosis with multiple mechanisms, including EMT transition^62–64^ and is known to be increased in BALF of SSc-ILD^65^ with immunohistochemical evaluations indicating alveolar macrophages and type 2 AECs as main producers^66^ and to be increased expression of TGF‑β target genes in SSc lung.^67^ Similarly, the evidence of IL-6 and TNF-α, and IL-8 hyperexpression in SSc-ILD lung and their ability to induce EMT in pre-clinical models^68–70^ furtherly support the pathophysiological hypothesis of an immune-induced EMT in SS-ILD.

### Micro-aspiration of gastric content as EMT-inducer in SSc-ILD

Gastro-oesophageal disease is an early and highly prevalent organ involvement of impaired distal oesophageal peristalsis, lower oesophageal sphincter pressure, reduced oesophageal acid clearance and gastroesophageal micro-aspiration.^71^ Of note, because of the multi-organ nature of SSc, the almost constant association of pulmonary interstitial fibrosis and oesophageal disease is a unicum in human pathology. The severity of ILD has been shown to be associated with the degree of oesophageal involvement so gastric micro-aspiration has been proposed as a key mechanism of the progression of lung fibrosis in SSc.^72^ Both acid and non-acid components of the reflux have been associated with worse pulmonary involvement. These clinical data are interestingly consistent with pre-clinical studies showing that EMT in lung tissue can be determined in animal models where pepsin,^73^ biliary acid^74^ or acid solution^75^ are delivered to the lungs via aspiration.

### Telomere dysfunction as EMT-inducer in SSc-ILD

The telomeres are complexes of DNA repeats and binding proteins that protect eukaryotic chromosomal ends, preserved across cellular divisions by the telomerase ribonucleoprotein and finally, prevent DNA damage responses and cellular senescence. Telomere dysfunction of AEC2s has been considered a key event in IPF pathogenesis,^76^ but also SSc patients were reported to show shorter age-standardized telomere length compared to healthy controls, especially in the presence of ILD.^77^ Nevertheless, one SSc patient in 10 is known to express autoantibodies that target telomere proteins and are associated with a more severe ILD and shorter telomere length.^78^

In preclinical models, telomere shortening enhances TGF-β-mediated pulmonary fibrosis after bleomycin exposure^79^ but the evidence about a direct pathogenetic connection between EMT and telomeres is still poor and conflicting. The telomerase has been shown to be downregulated by TGF-β through SNAIL1 in murine mesenchymal staminal cells^80^ but also to promote EMT in lung and oral cancer cells.^29,81^

## Could alveolar EMT be a future therapeutic target in SSc-ILD?

The licenced anti-fibrotic medications, namely nintedanib and pirfenidone, have shown *in vitr*o an ability to counteract EMT that could contribute to their therapeutic value together with the anti-proliferative effect towards fibroblast and, possibly, anti-angiogenetic effect. Furthermore, current immunosuppressive medications with an established efficacy in SSc-ILD, – namely cyclophosphamide, mycophenolate mofetil, tocilizumab and rituximab – are certainly able to address the multiple pro-inflammatory signals that have been demonstrated to be potent inducers of EMT. Nevertheless, medications such as azithromycin, atorvastatin and losartan showed anti-EMT effects with a demonstrated or postulated marginal clinical effect on lung fibrosis.^82^

Current efforts towards the development of drugs that are specifically able to interfere with EMT have been mainly made in cancer research with more than one-hundred drugs under investigation given the known involvement of the process in neoplastic progression. Different strategies have been proposed to counteract the process in different stages, but given the complexity and the interconnections of the involved pathways, it is often hard to distinguish between anti-EMT and anti-proliferative effects. These approaches include blockage of EMT upstream extracellular signals such as TGF-β or Wnt/β-catenin pathways, targeting EMT-related transcription factors including members of the SNAIL, TWIST and ZEB families, or even targeting the mesenchymal cells themselves by inhibiting the functions of mesenchymal-specific proteins such as vimentin, fibronectin and N-cadherin or their ability to interact with EMC components.^83^

Accumulating the evidence on the EMT role in SSc-ILD could pave the way for anti-EMT treatment also in this disease with EMT reversing as a specifically attractive therapeutic option. On the other side, SSc-ILD patients could be exposed to specific risks related to the interference with normal homeostatic processes and in particular negative effects on healing of skin ulcers. Nevertheless, patients and disease stages in which SSc-ILD patients would benefit the most from an anti-EMT therapy should be identified.

## Conclusion

While there is an abundance of pre-clinical and clinical evidence to support the role of EMT in SSc-ILD development and progression, two main research priorities should be addressed by the research agenda on the topic.

First, the effective contribution of EMT process to lung fibrosis in SSc should be demonstrated. As reported above, this evidence is quite hard to be derived from animal or cellular models and a direct characterization of SSc-ILD lung tissue is needed. Given the high mortality associated with lung involvement, the use of lung biopsies or BALF collection of selected patients for research purpose could provide definitive evidence on SSc-ILD pathophysiology and should be explored in future research proposals. Of note, both these procedures are already extensively used in IPF and lung-transplanted patients and are now safe and associated with a limited patient discomfort when performed in experienced centres. Nevertheless, considering that routinary resort to invasive procedures is not feasible in clinical practice, the investigation of serum markers of epithelial damage or activation, should be implemented to easily extend the inference driven from lung-derived samples to the largest number of SSc-ILD patients.

The second point of the research agenda should be focused on the efficacy assessment of the available medications against EMT in SSc-ILD patients. The preliminary evaluation of the potentially different role of EMT in different SSc-ILD patients could guide an enriched enrolment in randomized controlled trials based on clinical characteristics and validated EMT markers. Nevertheless, the possibility of SSc-specific adverse events that are less relevant in neoplastic patients should be considered, with a specific reference to digital ulceration and tissue healing.

Data consolidation on SSc-ILD EMT could in conclusion pave the way for new therapeutic opportunities to prevent, slow or even reverse lung fibrosis.

## References

[1] Suliman S Al Harash A Roberts WN , et al. Scleroderma-related interstitial lung disease. Respir Med Case Rep 2017; 22: 109–112. 28761806 10.1016/j.rmcr.2017.07.007 PMC5524221

[2] Takahashi M Kunugi S Terasaki Y , et al. The difference of neovascularization in early intra-alveolar fibrosis between nonspecific interstitial pneumonia and usual interstitial pneumonia. Pathol Int 2013; 63(5): 237–244. 23714250 10.1111/pin.12058

[3] Bouros D Wells AU Nicholson AG , et al. Histopathologic subsets of fibrosing alveolitis in patients with systemic sclerosis and their relationship to outcome. Am J Respir Crit Care Med 2002; 165(12): 1581–1586. 12070056 10.1164/rccm.2106012

[4] Konopka KE Myers JL . Interstitial lung disease pathology in systemic sclerosis. Ther Adv Musculoskelet Dis 2021; 13: 1759720X211032437. 10.1177/1759720X211032437 PMC8287363 34349846

[5] Willis BC duBois RM Borok Z . Epithelial origin of myofibroblasts during fibrosis in the lung. Proc Am Thorac Soc 2006; 3(4): 377–382. 16738204 10.1513/pats.200601-004TK PMC2658689

[6] Nieto MA . The ins and outs of the epithelial to mesenchymal transition in health and disease. Annu Rev Cell Dev Biol 2011; 27: 347–376. 21740232 10.1146/annurev-cellbio-092910-154036

[7] Kalluri R Weinberg RA . The basics of epithelial-mesenchymal transition. J Clin Invest 2009; 119(6): 1420–1428. 19487818 10.1172/JCI39104 PMC2689101

[8] Nieto MA Huang RY Jackson RA , et al. EMT: 2016 Cell 2016; 166(1): 21–45. 27368099 10.1016/j.cell.2016.06.028

[9] Mason RJ . Biology of alveolar type II cells. Respirology 2006; 11 Suppl: S12–S15. 10.1111/j.1440-1843.2006.00800.x 16423262

[10] Frye BC Schupp JC Rothe ME , et al. The value of bronchoalveolar lavage for discrimination between healthy and diseased individuals. J Intern Med 2020; 287(1): 54–65. 31612575 10.1111/joim.12973

[11] Borok Z Lubman RL Danto SI , et al. Keratinocyte growth factor modulates alveolar epithelial cell phenotype in vitro: expression of aquaporin 5. Am J Respir Cell Mol Biol 1998; 18(4): 554–561. 9533944 10.1165/ajrcmb.18.4.2838

[12] Lee JM Dedhar S Kalluri R , et al. The epithelial-mesenchymal transition: new insights in signalling, development, and disease. J Cell Biol 2006; 172(7): 973–981. 16567498 10.1083/jcb.200601018 PMC2063755

[13] Kage H Borok Z . EMT and interstitial lung disease: a mysterious relationship. Curr Opin Pulm Med 2012; 18(5): 517–523. 22854509 10.1097/MCP.0b013e3283566721 PMC3914631

[14] Luecken MD Theis FJ . Current best practices in single-cell RNA-seq analysis: a tutorial. Mol Syst Biol 2019; 15(6): e8746. 10.15252/msb.20188746 PMC6582955 31217225

[15] Kretzschmar K Watt FM . Lineage tracing. Cell 2012; 148(1–2): 33–45. 22265400 10.1016/j.cell.2012.01.002

[16] Kim KK Kugler MC Wolters PJ , et al. Alveolar epithelial cell mesenchymal transition develops in vivo during pulmonary fibrosis and is regulated by the extracellular matrix. Proc Natl Acad Sci U S A 2006; 103(35): 13180–13185. 16924102 10.1073/pnas.0605669103 PMC1551904

[bibr17-23971983231181727] Tanjore H Xu XC Polosukhin VV , et al. Contribution of epithelial-derived fibroblasts to bleomycin-induced lung fibrosis. Am J Respir Crit Care Med 2009; 180(7): 657–665. 19556518 10.1164/rccm.200903-0322OC PMC2753790

[18] Rock JR Barkauskas CE Cronce MJ , et al. Multiple stromal populations contribute to pulmonary fibrosis without evidence for epithelial to mesenchymal transition. Proc Natl Acad Sci U S A 2011; 108(52): E1475–E1483. 10.1073/pnas.1117988108 PMC3248478 22123957

[19] Tabuchi A Mertens M Kuppe H , et al. Intravital microscopy of the murine pulmonary microcirculation. J Appl Physiol 2008; 104(2): 338–346. 18006870 10.1152/japplphysiol.00348.2007

[20] Kowal-Bielecka O Kowal K Highland KB , et al. Bronchoalveolar lavage fluid in scleroderma interstitial lung disease: technical aspects and clinical correlations: a review of the literature. Semin Arthritis Rheum 2010; 40(1): 73–88. 19152959 10.1016/j.semarthrit.2008.10.009

[21] Marmai C Sutherland RE Kim KK , et al. Alveolar epithelial cells express mesenchymal proteins in patients with idiopathic pulmonary fibrosis. Am J Physiol Lung Cell Mol Physiol 2011; 301(1): L71–L78. 10.1152/ajplung.00212.2010 PMC3129898 21498628

[22] Ward C Forrest IA Murphy DM , et al. The phenotype of airway epithelial cells suggests epithelial to mesenchymal cell transition in clinically stable lung transplant recipients. Thorax 2005; 60(10): 865–871. 15972366 10.1136/thx.2005.043026 PMC1747194

[23] Valenzi E Tabib T Papazoglou A , et al. Disparate interferon signaling and shared aberrant basaloid cells in single-cell profiling of idiopathic pulmonary fibrosis and systemic sclerosis-associated interstitial lung disease. Front Immunol 2021; 12: 595811. 33859634 10.3389/fimmu.2021.595811 PMC8042271

[24] Hant FN Ludwicka-Bradley A Wang HJ , et al. Surfactant protein D and KL-6 as serum biomarkers of interstitial lung disease in patients with scleroderma. J Rheumatol 2009; 36(4): 773–780. 19286849 10.3899/jrheum.080633

[25] Wells AU Hansell DM Harrison NK , et al. Clearance of inhaled 99mTc-DTPA predicts the clinical course of fibrosing alveolitis. Eur Respir J 1993; 6(6): 797–802. 8339797

[26] Schniering J Guo L Brunner M , et al. Evaluation of 99mTc-rhAnnexin V-128 SPECT/CT as a diagnostic tool for early stages of interstitial lung disease associated with systemic sclerosis. Arthritis Res Ther 2018; 20(1): 183. 30115119 10.1186/s13075-018-1681-1 PMC6097327

[27] Hiemstra PS Tetley TD Janes SM . Airway and alveolar epithelial cells in culture. Eur Respir J 2019; 54(5): 1900742. 31515398 10.1183/13993003.00742-2019

[28] McGonigle P Ruggeri B . Animal models of human disease: challenges in enabling translation. Biochem Pharmacol 2014; 87(1): 162–171. 23954708 10.1016/j.bcp.2013.08.006

[29] Prasad RR Mishra DK Kumar M , et al. Human telomerase reverse transcriptase promotes the epithelial to mesenchymal transition in lung cancer cells by enhancing c-MET upregulation. Heliyon 2021; 8(1): e08673. 10.1016/j.heliyon.2021.e08673 PMC8732784 35024489

[30] Liu Z Li Q Li K , et al. Telomerase reverse transcriptase promotes epithelial-mesenchymal transition and stem cell-like traits in cancer cells. Oncogene 2013; 32(36): 4203–4213. 23045275 10.1038/onc.2012.441

[31] Degryse AL Tanjore H Xu XC , et al. TGFβ signaling in lung epithelium regulates bleomycin-induced alveolar injury and fibroblast recruitment. Am J Physiol Lung Cell Mol Physiol 2011; 300(6): L887–L897. 10.1152/ajplung.00397.2010 PMC3119129 21441353

[32] Kim KK Wei Y Szekeres C , et al. Epithelial cell alpha3beta1 integrin links beta-catenin and Smad signalling to promote myofibroblast formation and pulmonary fibrosis. J Clin Invest 2009; 119(1): 213–224. 19104148 10.1172/JCI36940 PMC2613463

[33] Tashiro J Rubio GA Limper AH , et al. Exploring animal models that resemble idiopathic pulmonary fibrosis. Front Med 2017; 4: 118. 10.3389/fmed.2017.00118 PMC5532376 28804709

[34] Yoshizaki A Iwata Y Komura K , et al. CD19 regulates skin and lung fibrosis via Toll-like receptor signalling in a model of bleomycin-induced scleroderma. Am J Pathol 2008; 172(6): 1650–1663. 18467694 10.2353/ajpath.2008.071049 PMC2408424

[35] Moeller A Ask K Warburton D , et al. The bleomycin animal model: a useful tool to investigate treatment options for idiopathic pulmonary fibrosis? Int J Biochem Cell Biol 2008; 40(3): 362–382. 17936056 10.1016/j.biocel.2007.08.011 PMC2323681

[36] Castaneda J Lesaux C Calhoun C , et al. Effect of mycophenolate in a mouse model of lung fibrosis. Chest 2017; 152(4): A489.

[37] Pignone A Scaletti C Matucci-Cerinic M , et al. Anti-endothelial cell antibodies in systemic sclerosis: significant association with vascular involvement and alveolo-capillary impairment. Clin Exp Rheumatol 1998; 16(5): 527–532. 9779298

[38] Trojanowska M . Cellular and molecular aspects of vascular dysfunction in systemic sclerosis. Nat Rev Rheumatol 2010; 6(8): 453–460. 20585340 10.1038/nrrheum.2010.102 PMC3824624

[39] Mendoza FA Piera-Velazquez S Farber JL , et al. Endothelial cells expressing endothelial and mesenchymal cell gene products in lung tissue from patients with systemic sclerosis-associated interstitial lung disease. Arthritis Rheumatol 2016; 68(1): 210–217. 26360820 10.1002/art.39421 PMC4690777

[40] Jimenez SA Piera-Velazquez S . Endothelial to mesenchymal transition (EndoMT) in the pathogenesis of systemic sclerosis-associated pulmonary fibrosis and pulmonary arterial hypertension. Matrix Biol 2016; 51: 26–36. 26807760 10.1016/j.matbio.2016.01.012 PMC4842122

[41] Khimdas S Harding S Bonner A , et al. Associations with digital ulcers in a large cohort of systemic sclerosis: results from the Canadian scleroderma research group registry. Arthritis Care Res 2011; 63(1): 142–149. 10.1002/acr.20336 20740608

[42] Umashankar E Abdel-Shaheed C Plit M , et al. Assessing the role for nailfold videocapillaroscopy in interstitial lung disease classification: a systematic review and meta-analysis. Rheumatology 2022; 61(6): 2221–2234. 34668513 10.1093/rheumatology/keab772

[43] Ihn H Sato S Fujimoto M , et al. Characterization of autoantibodies to endothelial cells in systemic sclerosis (SSc): association with pulmonary fibrosis. Clin Exp Immunol 2000; 119(1): 203–209. 10606984 10.1046/j.1365-2249.2000.01115.x PMC1905540

[44] Fields A Potel KN Cabuhal R , et al. Mediators of systemic sclerosis-associated interstitial lung disease (SSc-ILD): systematic review and meta-analyses. Thorax. Epub ahead of print 19 October 2022. DOI: 10.1136/thorax-2022-219226. PMC10359532 36261273

[45] Matucci-Cerinic M Pignone A Iannone F , et al. Clinical correlations of plasma angiotensin converting enzyme (ACE) activity in systemic sclerosis: a longitudinal study of plasma ACE level, endothelial injury and lung involvement. Respir Med 1990; 84(4): 283–287. 2173047 10.1016/s0954-6111(08)80054-6

[46] Cambrey AD Harrison NK Dawes KE , et al. Increased levels of endothelin-1 in bronchoalveolar lavage fluid from patients with systemic sclerosis contribute to fibroblast mitogenic activity in vitro. Am J Respir Cell Mol Biol 1994; 11(4): 439–445. 7917311 10.1165/ajrcmb.11.4.7917311

[47] Ohba T McDonald JK Silver RM , et al. Scleroderma bronchoalveolar lavage fluid contains thrombin, a mediator of human lung fibroblast proliferation via induction of platelet-derived growth factor alpha-receptor. Am J Respir Cell Mol Biol 1994; 10(4): 405–412. 7510986 10.1165/ajrcmb.10.4.7510986

[48] Odoux C Crestani B Lebrun G , et al. Endothelin-1 secretion by alveolar macrophages in systemic sclerosis. Am J Respir Crit Care Med 1997; 156(5): 1429–1435. 9372656 10.1164/ajrccm.156.5.96-11004

[49] Fagan KA McMurtry IF Rodman DM . Role of endothelin-1 in lung disease. Respir Res 2001; 2(2): 90–101. 11686871 10.1186/rr44 PMC59574

[50] Gorowiec MR Borthwick LA Parker SM , et al. Free radical generation induces epithelial-to-mesenchymal transition in lung epithelium via a TGF-β1-dependent mechanism. Free Radic Biol Med 2012; 52(6): 1024–1032. 22240154 10.1016/j.freeradbiomed.2011.12.020

[bibr51-23971983231181727] Felton VM Borok Z Willis BC . N-acetylcysteine inhibits alveolar epithelial-mesenchymal transition. Am J Physiol Lung Cell Mol Physiol 2009; 297(5): L805–L812. 10.1152/ajplung.00009.2009 PMC2777496 19648289

[52] Zhou G Dada LA Wu M , et al. Hypoxia-induced alveolar epithelial-mesenchymal transition requires mitochondrial ROS and hypoxia-inducible factor 1. Am J Physiol Lung Cell Mol Physiol 2009; 297(6): L1120–L1130. 10.1152/ajplung.00007.2009 PMC2793183 19801454

[53] Vyas-Read S Shaul PW Yuhanna IS , et al. Nitric oxide attenuates epithelial-mesenchymal transition in alveolar epithelial cells. Am J Physiol Lung Cell Mol Physiol 2007; 293(1): L212–L221. 10.1152/ajplung.00475.2006 17496059

[54] Jain R Shaul PW Borok Z , et al. Endothelin-1 induces alveolar epithelial-mesenchymal transition through endothelin type A receptor-mediated production of TGF-beta1. Am J Respir Cell Mol Biol 2007; 37(1): 38–47. 17379848 10.1165/rcmb.2006-0353OC PMC1899351

[55] Howell DC Goldsack NR Marshall RP , et al. Direct thrombin inhibition reduces lung collagen, accumulation, and connective tissue growth factor mRNA levels in bleomycin-induced pulmonary fibrosis. Am J Pathol 2001; 159(4): 1383–1395. 11583966 10.1016/S0002-9440(10)62525-4 PMC1850500

[56] Heukels P Moor CC von der Thüsen JH , et al. Inflammation and immunity in IPF pathogenesis and treatment. Respir Med 2019; 147: 79–91. 30704705 10.1016/j.rmed.2018.12.015

[57] da Silva SO da Paz AS Farias IMVC , et al. Bronchoalveolar lavage in systemic sclerosis patients: a systematic review. Curr Rheumatol Rev 2021; 17(2): 176–183. 33185168 10.2174/1573397116666201113091655

[58] Mathai SK Gulati M Peng X , et al. Circulating monocytes from systemic sclerosis patients with interstitial lung disease show an enhanced profibrotic phenotype. Lab Invest 2010; 90(6): 812–823. 20404807 10.1038/labinvest.2010.73 PMC3682419

[59] Murray LA Rosada R Moreira AP , et al. Serum amyloid P therapeutically attenuates murine bleomycin-induced pulmonary fibrosis via its effects on macrophages. PLoS ONE 2010; 5(3): e9683. 10.1371/journal.pone.0009683 PMC2837381 20300636

[60] Zhu L Fu X Chen X , et al. M2 macrophages induce EMT through the TGF-β/Smad2 signaling pathway. Cell Biol Int 2017; 41(9): 960–968. 28493530 10.1002/cbin.10788

[61] Homer RJ Herzog EL . Recent advances in pulmonary fibrosis: implications for scleroderma. Curr Opin Rheumatol 2010; 22(6): 683–689. 20693906 10.1097/BOR.0b013e32833ddcc9

[62] Li M Krishnaveni MS Li C , et al. Epithelium-specific deletion of TGF-β receptor type II protects mice from bleomycin-induced pulmonary fibrosis. J Clin Invest 2011; 121(1): 277–287. 21135509 10.1172/JCI42090 PMC3007138

[bibr63-23971983231181727] Kasai H Allen JT Mason RM , et al. TGF-beta1 induces human alveolar epithelial to mesenchymal cell transition (EMT). Respir Res 2005; 6(1): 56. 15946381 10.1186/1465-9921-6-56 PMC1177991

[64] Willis BC Liebler JM Luby-Phelps K , et al. Induction of epithelial-mesenchymal transition in alveolar epithelial cells by transforming growth factor-beta1: potential role in idiopathic pulmonary fibrosis. Am J Pathol 2005; 166(5): 1321–1332. 15855634 10.1016/s0002-9440(10)62351-6 PMC1606388

[65] Ludwicka A Ohba T Trojanowska M , et al. Elevated levels of platelet-derived growth factor and transforming growth factor-beta 1 in bronchoalveolar lavage fluid from patients with scleroderma. J Rheumatol 1995; 22(10): 1876–1883. 8991985

[66] Corrin B Butcher D McAnulty BJ , et al. Immunohistochemical localization of transforming growth factor-beta 1 in the lungs of patients with systemic sclerosis, cryptogenic fibrosing alveolitis and other lung disorders. Histopathology 1994; 24(2): 145–150. 8181807 10.1111/j.1365-2559.1994.tb01293.x

[67] Christmann RB Sampaio-Barros P Stifano G , et al. Association of Interferon- and transforming growth factor β-regulated genes and macrophage activation with systemic sclerosis-related progressive lung fibrosis. Arthritis Rheumatol 2014; 66(3): 714–725. 24574232 10.1002/art.38288 PMC4439004

[68] Liu Y Hu M Fan G , et al. Effect of Baricitinib on the epithelial-mesenchymal transition of alveolar epithelial cells induced by IL-6. Int Immunopharmacol 2022; 110: 109044. 35850052 10.1016/j.intimp.2022.109044

[bibr69-23971983231181727] Yamauchi Y Kohyama T Takizawa H , et al. Tumor necrosis factor-alpha enhances both epithelial-mesenchymal transition and cell contraction induced in A549 human alveolar epithelial cells by transforming growth factor-beta1. Exp Lung Res 2010; 36(1): 12–24. 20128678 10.3109/01902140903042589

[70] Desai S Laskar S Pandey BN . Autocrine IL-8 and VEGF mediate epithelial-mesenchymal transition and invasiveness via p38/JNK-ATF-2 signalling in A549 lung cancer cells. Cell Signal 2013; 25(9): 1780–1791. 23714383 10.1016/j.cellsig.2013.05.025

[71] McFarlane IM Bhamra MS Kreps A , et al. Gastrointestinal manifestations of systemic sclerosis. Rheumatology 2018; 8(1): 235. 30057856 10.4172/2161-1149.1000235 PMC6059963

[72] Christmann RB Wells AU Capelozzi VL , et al. Gastroesophageal reflux incites interstitial lung disease in systemic sclerosis: clinical, radiologic, histopathologic, and treatment evidence. Semin Arthritis Rheum 2010; 40(3): 241–249. 20494406 10.1016/j.semarthrit.2010.03.002

[73] Ahmad F Ritzenthaler J Roman J . Pepsin in gastric fluid promotes epithelial mesenchymal transformation: implications for understanding the role of reflux in pulmonary fibrosis. Am J Respir Crit Care Med 2009; 179: A5311.

[74] Chen B You WJ Liu XQ , et al. Chronic microaspiration of bile acids induces lung fibrosis through multiple mechanisms in rats. Clin Sci 2017; 131(10): 951–963. 10.1042/CS20160926 28341659

[75] Cabrera-Benítez NE Parotto M Post M , et al. Mechanical stress induces lung fibrosis by epithelial-mesenchymal transition. Crit Care Med 2012; 40(2): 510–517. 21926573 10.1097/CCM.0b013e31822f09d7 PMC5061566

[76] Zhang K Xu L Cong YS . Telomere dysfunction in idiopathic pulmonary fibrosis. Front Med 2021; 8: 739810. 10.3389/fmed.2021.739810 PMC8631932 34859008

[77] Usategui A Municio C Arias-Salgado EG , et al. Evidence of telomere attrition and a potential role for DNA damage in systemic sclerosis. Immun Ageing 2022; 19(1): 7. 35086525 10.1186/s12979-022-00263-2 PMC8793167

[78] Adler BL Boin F Wolters PJ , et al. Autoantibodies targeting telomere-associated proteins in systemic sclerosis. Ann Rheum Dis 2021; 80(7): 912–919. 33495152 10.1136/annrheumdis-2020-218918 PMC8217217

[79] Liu YY Shi Y Liu Y , et al. Telomere shortening activates TGF-β/Smads signaling in lungs and enhances both lipopolysaccharide and bleomycin-induced pulmonary fibrosis. Acta Pharmacol Sin 2018; 39(11): 1735–1745. 29925920 10.1038/s41401-018-0007-9 PMC6289325

[80] Mazzolini R Gonzàlez N Garcia-Garijo A , et al. Snail1 transcription factor controls telomere transcription and integrity. Nucleic Acids Res 2018; 46(1): 146–158. 29059385 10.1093/nar/gkx958 PMC5758914

[81] Zhao T Hu F Qiao B , et al. Telomerase reverse transcriptase potentially promotes the progression of oral squamous cell carcinoma through induction of epithelial-mesenchymal transition. Int J Oncol 2015; 46(5): 2205–2215. 25775973 10.3892/ijo.2015.2927

[82] Jolly MK Ward C Eapen MS , et al. Epithelial-mesenchymal transition, a spectrum of states: role in lung development, homeostasis, and disease. Dev Dyn 2018; 247(3): 346–358. 28646553 10.1002/dvdy.24541

[83] Jonckheere S Adams J De Groote D , et al. Epithelial-mesenchymal transition (EMT) as a therapeutic target. Cells Tissues Organs 2022; 211(2): 157–182. 33401271 10.1159/000512218

